# Five screening-detected breast cancer cases in initially disease-free *BRCA1* or *BRCA2* mutation carriers

**DOI:** 10.1007/s12282-019-00971-6

**Published:** 2019-04-12

**Authors:** Satoko Shimada, Reiko Yoshida, Eri Nakashima, Dai Kitagawa, Naoya Gomi, Rie Horii, Sayoko Takeuchi, Yuumi Ashihara, Mizuho Kita, Futoshi Akiyama, Shinji Ohno, Mitsue Saito, Masami Arai

**Affiliations:** 1 Division of Clinical Genetic Oncology, Cancer Institute Hospital, Japanese Foundation for Cancer Research, 3-8-31 Ariake Koto-ku, Tokyo, 135-8550 Japan; 2 Department of Breast Oncology, Juntendo University, Tokyo, Japan; 3 Breast Oncology Center, Cancer Institute Hospital, Japanese Foundation for Cancer Research, Tokyo, Japan; 4 Diagnostic Imaging Center, Cancer Institute Hospital, Japanese Foundation for Cancer Research, Tokyo, Japan; 5 Department of Pathology, Cancer Institute Hospital, Japanese Foundation for Cancer Research, Tokyo, Japan; 6 Department of Pathology, Cancer Institute, Japanese Foundation for Cancer Research, Tokyo, Japan; 7 Diagnostics and Therapeutics of Intractable Disease, Graduate School of Medicine, Juntendo University, Tokyo, Japan

**Keywords:** *BRCA1* mutation carrier, *BRCA2* mutation carrier, Screening-detected breast cancer, Breast cancer incidence

## Abstract

Individuals carrying pathogenic *BRCA1* or *BRCA2* mutations have an increased lifetime risk of breast and/or ovarian cancer. The incidence of breast cancer amongst disease-free *BRCA* mutation carriers under surveillance and the clinical and pathological characteristics of those who subsequently develop the disease remain unclear in Japan. We reviewed the records of 155 individuals with *BRCA1* or *BRCA2* mutations identified by genetic testing between January 2000 and December 2016. At the time of genetic testing, 26 individuals with one of these mutations had no history of breast cancer and were therefore enrolled in a surveillance program that included biannual ultrasonography, clinical breast examination, annual mammography, and conditional magnetic resonance imaging for the early detection of primary breast cancer. During the surveillance period, 5 individuals with *BRCA1* or *BRCA2* mutations were diagnosed with primary breast cancer. The mean surveillance duration until breast cancer diagnosis was 48 months. The incidence of primary breast cancer during surveillance in initially disease-free *BRCA* mutation carriers was 4.23%/year. In two cases, the tumors were only detectable on MRI. The case 5 patient who presented with a tumor that was detected by self-examination, which then grew rapidly, had stage IIB triple-negative breast cancer. In conclusion, our results show that some challenges exist in the early detection of breast cancers in *BRCA1* or *BRCA2* mutation carriers. There are also some difficulties in approaching those individuals in Japanese society.

## Introduction

Clinical management of hereditary breast and ovarian cancer (HBOC) is important as these women are at higher risk of developing breast cancer. The breast cancer incidence rate peaks at an earlier age, i.e., in the late 40s, in Japan than in western countries. Women with HBOC are diagnosed at an even younger age and often during important phases of their lives, such as the period of childbirth and rearing. The implementation of early detection and prevention strategies thus plays an important societal role. Risk-reduction mastectomy has not only reduced the incidence of contralateral breast cancer but has also improved the overall survival rate. In a clinical setting, the identification of *BRCA1* or *BRCA2* mutations has gained significance as a tool for the implementation of cancer risk-reducing strategies. We describe herein the clinicopathological characteristics in surveillance-detected breast cancers among unaffected *BRCA* mutation carriers.

## Patients and methods

Between January 2000 and December 2016, 776 probands visited the outpatient division of the clinical genetic oncology department at the Cancer Institute Hospital (CIH) in Tokyo. Of these, 550 (464 probands and 86 family members) underwent genetic testing. *BRCA1* or *BRCA2* mutation was detected in 155 individuals, including suspected deleterious mutations identified by genetic testing. At the time of receiving their genetic test results, 26 individuals with one of these mutations were disease-free, but had a family history of at least breast cancer or ovarian cancer. Those 26 individuals were enrolled in an intensive surveillance protocol that included biannual ultrasonography and clinical breast examination (CBE), annual mammography (MMG), and conditional contrast-enhanced magnetic resonance imaging (MRI) for the early detection of primary breast cancer in the CIH in Tokyo. Observation of these individuals started from the day of the release of genetic testing results until either the day of primary breast cancer diagnosis or the end of 2017. This study was approved by the Institutional Review Board of CIH (2016-1151), and informed consent was obtained from all subjects.

## Results

Five of the 26 individuals with *BRCA1* or *BRCA2* mutations were diagnosed with primary breast cancer while undergoing surveillance. Table 1 shows the baseline and clinical characteristics of the subjects included in this study. The mean age at genetic testing of the five patients who were diagnosed with breast cancer was lower than that of those who remained breast cancer-free (37.5 years vs 46.1 years). The mean duration of surveillance until breast cancer diagnosis was 48 months. Regarding genotype, two cases had a *BRCA1* mutation and three had a *BRCA2* mutation. Three out of five cases had only ductal carcinoma in situ (DCIS); one of these cases had irregular calcifications detected on MMG, and the other two had tumors that were detectable only by MRI. The five cases of breast cancer in previously unaffected *BRCA* mutation carriers are described below in further detail.

**Table 1:** Baseline and clinical characteristics of the subjects included in this study

Characteristics	Breast cancer patients (*n* = 5)	Subjects without breast cancer (*n* = 21)
Mean age at diagnosis/end of 2017 (years)	41.4 (35–48)	48 (28–76)
Mean age at genetic testing (years)	37.5 (23–46)	46.1 (25–71)
Mean duration of surveillance until cancer diagnosis/end of 2017 (months)	48 (4–145)	56 (19–100)
Mutation		
*BRCA1*	2 (40)	16 (76.2)
*BRCA2*	3 (60)	5 (23.8)
Salpingo-oophorectomy		
Yes	0	8 (38.1)
No	5 (100)	13 (61.9)
Family history of breast cancer		
Yes	5 (100)	18 (85.7)
No	0	3 (14.3)
Age of the youngest family member at breast cancer diagnosis (years)	30	25
Family history of ovarian cancer		
Yes	1 (20)	15 (71.4)
No	4 (80)	6 (28.6)
Age of the youngest family at ovarian cancer diagnosis (years)	33	40
Ovarian cancer diagnosis		
Yes	0	3 (14.3)
No	5 (100)	18 (85.7)

Data are given as mean values (range) or *n* (%). Mean age was calculated using age at diagnosis for those with breast cancer, or age on 31/12/2017 for those without

### Case 1

A 42-year-old woman with a *BRCA1* mutation developed DCIS as a primary breast cancer during surveillance only 4 months after genetic testing. She had received regular check-ups every year for 7 years. She had a positive family history of breast cancer: her sisters were diagnosed with breast cancer at 38 and 45 years of age, and her paternal grandmother was diagnosed at 70 years of age. Cancer was detected by MMG examination. She underwent total mastectomy and sentinel lymph node biopsy, and the tumor was shown to be pathological stage 0.

### Case 2

A 35-year-old woman with a *BRCA1* mutation developed DCIS as a primary breast cancer during surveillance, 12 years after genetic testing was performed. She had a strong positive family history of breast cancer: her mother had bilateral breast cancer, with one tumor diagnosed at 45 years of age and the other at 48 years of age; her maternal grandmother had been diagnosed with breast cancer at age 90 years of age; and her maternal aunt’s daughter had breast and ovarian cancer at 33 years of age. Furthermore, the patient’s mother was diagnosed with ovarian cancer at 62 years of age. This patient had undergone breast screening examination every 6 months. Her cancer was detected only on MRI screening. The lesion was described as a non-mass with high signal on diffusion-weighed imaging (DWI). The apparent diffusion coefficient (ADC) level was slightly reduced. She underwent a total mastectomy and sentinel lymph node biopsy. The tumor was pathological stage 0 (Table 2).

**Table 2:** Characteristics of the 5 cases diagnosed with breast cancer during the surveillance period

Patient no.	Age at breast cancer diagnosis (years)	Mutation	Surveillance period (months)	Diagnostic modality	Family history	Pathology	Stage	Outcome
1	42	*BRCA1*	4	MMG	BC	DCIS	TisN0M0 Stage 0	Alive without metastasis
2	35	*BRCA1*	145	MRI	BC+OC	DCIS	TisN0M0 Stage 0	Alive without metastasis
3	47	*BRCA2*	7	US	BC	IDC	T1N0M0 StageI	Alive without metastasis
4	48	*BRCA2*	51	MRI	BC	DCIS	TisN0M0 Stage 0	Alive without metastasis
5	35	*BRCA2*	35	CE (self-palpation)	BC+OC	IDC	T2N1M0 Stage IIB	Alive without metastasis

*MMG* mammography, *US* ultrasonography, *MRI* magnetic resonance imaging, *CE* clinical examination, *BC* breast cancer, *OC* ovarian cancer

### Case 3

A 47-year-old woman with a *BRCA2* mutation developed a 4-mm-sized invasive ductal carcinoma (IDC) that was estrogen receptor (ER)-positive and human epidermal growth factor receptor (HER2)-negative. The carcinoma developed as a primary breast cancer during the surveillance period 7 months after undergoing genetic testing. Her cancer was first detected by ultrasonography. She had a positive family history of breast cancer: her monozygotic twin sister had breast cancer at 44 years of age, and her paternal grandmother had been diagnosed in her 50s. The patient underwent total mastectomy and sentinel lymph node biopsy; the tumor was pathological stage I.

### Case 4

A 48-year-old woman with a *BRCA2* mutation developed DCIS as a primary breast cancer during surveillance 3 years after genetic testing. She had a strong positive family history of breast cancer: her mother had been diagnosed with breast cancer at 53 years of age, her maternal aunt at 52 years of age, one of her sisters at 30 years of age, and her youngest sister at 36 years of age. This patient had undergone breast examinations every 6 months. Her cancer was detected only on MRI. The lesion was also described as a non-mass-like enhancement in contrast-enhanced medium but was not detectable on DWI. She underwent a total mastectomy and sentinel lymph node biopsy. The tumor was pathological stage 0.

### Case 5

**Fig. 1:**
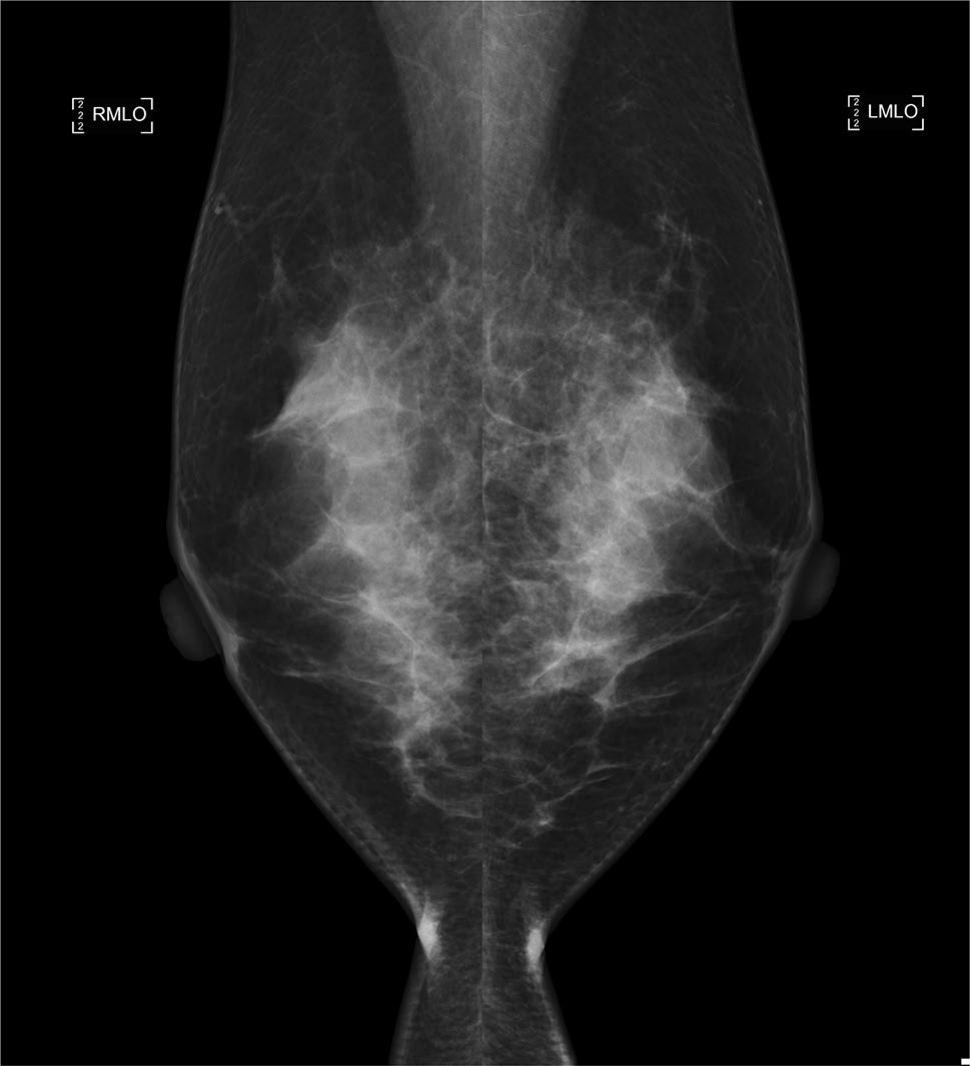
Mammography examination of case 5 revealed a possible focal asymmetric density on the upper area of the right breast with mediolateral oblique (MLO) view. *RMLO* right mediolateral oblique, *LMLO* left mediolateral oblique

**Fig. 2:**
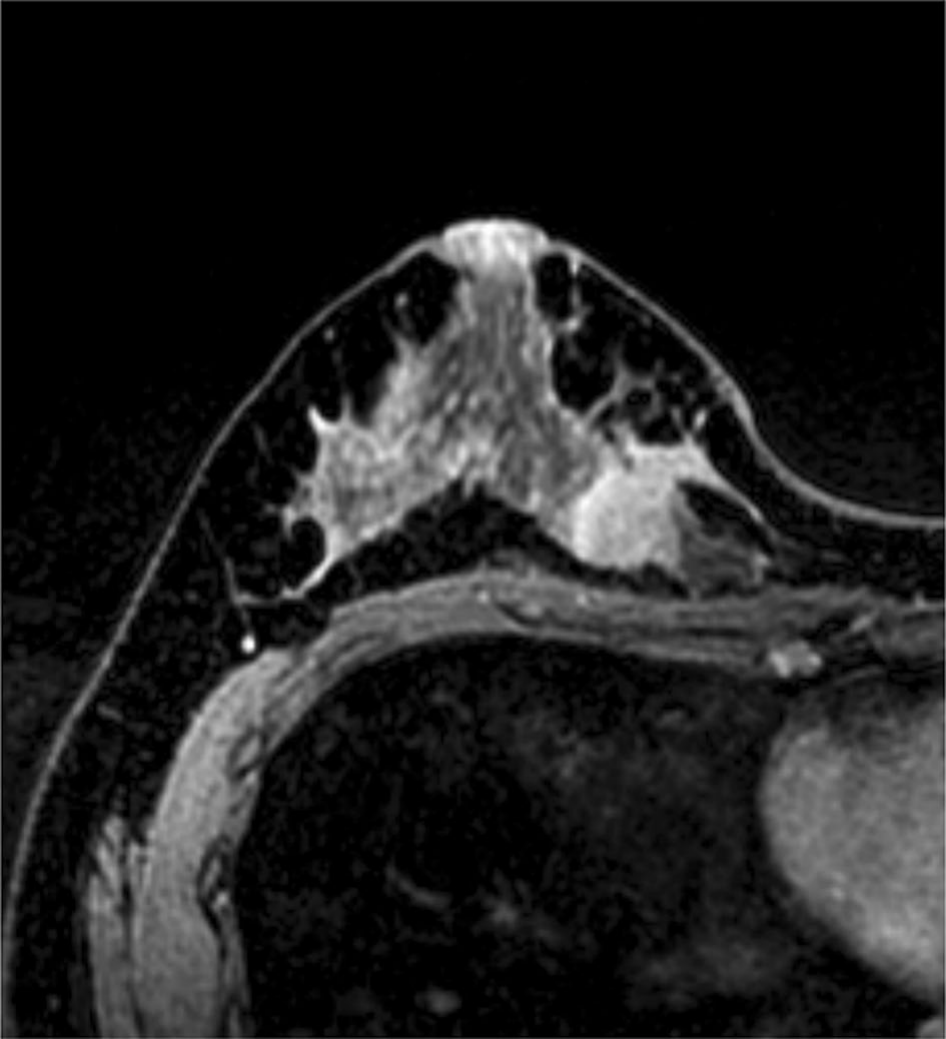
Contrast-enhanced magnetic resonance imaging in case 5 revealed a partially enhanced irregular mass of 3.2 cm in the upper-inner area of the right breast

A 35-year-old woman with a *BRCA2* mutation developed IDC that was ER-, progesterone receptor (PgR)-negative, and HER2-negative, as a primary breast cancer during surveillance, 2 years after receiving her genetic testing results. She had a strong positive family history of breast cancer: her mother had bilateral breast cancer, with one tumor diagnosed at 47 years of age and the other at 49 years of age; her maternal aunt also had bilateral breast cancer, with one tumor diagnosed at 45 years of age and the other at 47 years of age. The patient presented with a tumor that had been detected by self-examination and which then grew rapidly in the interim. Figures 1 and 2 show the findings on MMG and MRI at the time of tumor detection, respectively. The lesion was described as a mass with high signal intensity in DWI of MRI. The ADC was reduced. However, there were no remarkable findings on MMG, ultrasonography, or CBE at the last evaluation, performed 3 months earlier. MRI was not performed at the last evaluation but had been performed the year before. She underwent a nipple-sparing mastectomy and axillary lymph node dissection; the tumor was pathological stage IIB.

## Discussion

According to the National Comprehensive Cancer Network guidelines [1] and the Japanese Breast Cancer Society guidelines [2], annual MRI should be performed as part of regular check-ups for individuals with *BRCA1* or *BRCA2* mutations. Annual MRI combined with MMG examination has been reported to be more effective than MMG, ultrasonography, or CBE alone for detecting malignancies in *BRCA1* and *BRCA2* mutation carriers [3–5]. MRI increases the sensitivity of breast cancer detection at earlier stages and facilitates early detection of familial breast cancer regardless of the patient’s age, breast density, or mutation status [6]. It was not possible to perform annual MRI examination in all our patients due to the costs, limited access to the device, or constitutional factors. In fact, cases 1 and 3 were diagnosed with breast cancer within a year from the commencement of intensive surveillance, before scheduled MRI was performed. In the other 3 breast cancer cases, the first MRI examination performed in these patients resulted in detection of DCIS in 2. In agreement with studies from other countries [3–5], the combination of MRI and MMG was associated with a diagnosis of cancer at an earlier stage in women with a *BRCA1* or *BRCA2* mutation undergoing surveillance.

The Consortium of Investigators of Modifiers of *BRCA1/2* stated that ER-negative cancers occurring in both *BRCA1* and *BRCA2* mutation carriers were associated with a higher histological grade than ER-positive cancers. Sixty-eight percent of breast cancers occurring in *BRCA1* carriers were triple-negative, compared to only 16% of breast cancers in *BRCA2* carriers [7]. In our study, the patient of case 5, who had a *BRCA2* mutation, presented with advanced breast cancer of nuclear grade 3 and a triple-negative subtype. There were no remarkable findings on MMG, ultrasonography, or CBE at the last evaluation, 3 months prior to diagnosis. MRI was not performed at the last evaluation but was performed in the previous year. At the time of breast cancer diagnosis, she had been under surveillance for 2 years. Early detection of breast cancer was difficult in this patient who was of child-bearing age, although she was highly aware of her risks. Women with *BRCA1* or *BRCA2* mutations strongly expressed preferences for breast cancer risk reduction and preservation of fertility [8].

Secondary breast cancers are mostly diagnosed at a more favorable stage than primary tumors [9]. Even when the secondary cancer was detected at a relatively early stage, the survival of patients with sporadic bilateral breast cancer was poorer than that of women with unilateral breast cancer [9–11]. If the interval between the primary surgery and contralateral breast cancer is short, the risk of relapse is greater and the breast cancer-specific and all-cause mortalities are higher [10, 12]. In case 5, the primary diagnosis was made at age 35, and both the patient’s mother and her maternal aunt had developed bilateral breast cancers with a 2-year interval. Contralateral risk-reducing mastectomy (CRRM) was offered to this patient. However, as she was of a child-bearing age, this made the decision to undergo CRRM very difficult.

Bilateral risk-reducing mastectomy (BRRM) reportedly reduces the risk of breast cancer significantly, although it does not completely eliminate the risk because of the possibility of residual mammary-gland tissue [13–15]. BRRM has not been proven to show improved survival rates [16]. Moreover, selecting appropriate high-risk cases for BRRM is difficult. Metcalfe et al suggested that women with *BRCA1* or *BRCA2* mutations who were treated for stage I or II breast cancer with bilateral mastectomy had better outcomes than those undergoing unilateral mastectomy [17]. According to a Dutch study, the overall 10-year contralateral breast cancer risk for unselected *BRCA1* or *BRCA2* mutation carriers is approximately 18%, whereas that for mutation carriers with a family history of breast cancer is higher, at 25% [18]. This study also emphasized the importance of age when the primary breast cancer is diagnosed, because in patients who were diagnosed with breast cancer before the age of 41 years, the 10-year contralateral breast cancer risk was 23.9%, compared to 12.6% for those aged 41–49 years [12]. The mean age at the primary breast cancer diagnosis in our five cases was 41.4 years, and the approaches to our cases should be carefully revised.

The current study has several limitations. First, this was a retrospective study. Additionally, the follow-up period was short, and annual MRI was not performed in all subjects. A longer follow-up period will be necessary in future studies to confirm our current findings.

In conclusion, we presented five cases of primary breast cancer in patients carrying a *BRCA1* or *BRCA2* mutation, whose tumors were diagnosed during surveillance. During the surveillance period, 5 individuals with *BRCA1* or *BRCA2* mutations were diagnosed with primary breast cancer. The mean surveillance duration until breast cancer diagnosis was 48 months. The incidence of primary breast cancer during surveillance in initially disease-free *BRCA* mutation carriers was 4.23%/year. Our results suggest that there are challenges involved in the early detection of breast cancers in *BRCA1* or *BRCA2* mutation carriers.
